# Delayed Matter

**Published:** 1896-05

**Authors:** 


### DELAYED MATTER.

The pressure for space has, to our regret, forced the holding over of communications in type. Among these has been the report of the "Golden Anniversary" published in this number. We are still obliged to defer other valuable matter.

